# Cause for concern: Resident experience in operative trauma during general surgery residency at a Canadian centre

**DOI:** 10.36834/cmej.69323

**Published:** 2020-12-07

**Authors:** Paul T. Engels, Andrew Versolatto, Qian Shi, Angela Coates, Timothy J. Rice

**Affiliations:** 1 Department of Surgery, McMaster University, Ontario, Canada; 2 Trauma Program, Hamilton General Hospital, Hamilton Health Sciences, Ontario, Canada

## Abstract

**Background:**

The ability to provide competent operative trauma care is a core objective of general surgery training but recent publications question the ability of graduates to meet this standard. To assess the adequacy of operative trauma exposure during residency, we constructed and analyzed a retrospective trauma operative case log for general surgery residents at a Canadian trauma centre.

**Methods:**

The Hamilton General Hospital Trauma Registry was used to identify all patients from July 2008 to June 2018 who underwent a trauma operation on the neck, chest, or abdomen. Medical records were reviewed to determine procedure type and resident presence.

**Results:**

In our study, 417 patients underwent 570 operations (422 abdominal, 103 thoracic, and 45 neck). For the 35 residents that completed their general surgery residency during the study, the median number of trauma laparotomies was 5, with only 14/35 (40%) present for ≥10 trauma operations. Only 10 residents (29%) were exposed to a neck exploration and 18 (51%) exposed to a thoracic operation for trauma.

**Conclusions:**

Operative trauma exposure amongst general surgery residents at an academic Canadian trauma centre was limited. Cumulative operative trauma surgery exposure of a typical graduating resident was inadequate when compared to Canadian and American accrediting-body standards.

## Abstract (fr)

**Contexte:**

La capacité d’offrir des soins de qualité en traumatisme opératoire est un objectif principal de la formation en chirurgie générale, mais des publications récentes contestent la capacité des diplômés à satisfaire cette norme. Pour évaluer le caractère adéquat de l’exposition à des traumatismes opératoires pendant la résidence, nous avons construit et analysé un registre rétrospectif des cas opératoires traumatologiques des résidents en chirurgie générale à un centre canadien de traumatologie.

**Méthodes:**

Le registre des traumatismes du Hamilton General Hospital a été utilisé pour identifier tous les patients de juillet 2008 à juin 2018 qui ont subi une chirurgie traumatologique au cou, au thorax ou à l’abdomen. Les dossiers médicaux ont été examinés pour établir le type de procédure et la présence de résidents.

**Résultats:**

Dans notre étude, 417 patients ont subi 570 opérations (422 à l’abdomen, 103 au thorax et 45 au cou). Pour les 35 résidents qui ont terminé leur résidence en chirurgie générale au cours de l’étude, le nombre médian de laparotomies traumatologiques a été de cinq, avec seulement 14/35 (40 %) présents pour dix opérations traumatologiques ou plus. Seulement 10 résidents (9 %) ont assisté à une exploration du cou et 18 (51 %) ont assisté à une chirurgie thoracique pour un trauma.

**Conclusions:**

L’exposition aux traumatismes opératoires chez les résidents en chirurgie générale à un centre universitaire canadien de traumatologie a été limitée. L’exposition cumulative à des chirurgies traumatologiques opératoires d’un résident diplômé type était inadéquate comparativement aux normes d’agrément des organismes canadiens et américains.

## Background

In 2016, Strumwasser et al. presented their study examining Accreditation Council for Graduate Medical Education (ACGME) general surgery residency case logs in the United States (US).^1^ In this study the authors concluded that “even with fellowship training, the graduating trauma/critical care fellow is still only as experienced in open trauma surgery as a general surgery resident who graduated 15 years ago.” Given the similar training paradigm in Canada, this concerning publication stimulated the present study’s authors to examine the current state of Canadian residency trauma training.^2^

Trauma care has been steadily moving toward more non-operative and less-invasive treatment strategies, thus steadily reducing the volume of operative trauma cases available for training.^3^ Nevertheless, competency in operative trauma care has remained a core objective within general surgery training as outlined by the Royal College of Physicians and Surgeons of Canada (RCPSC).^4^ In addition, the training of general surgeons in Canada is currently undergoing massive change with the introduction of the Competency By Design (CBD) training paradigm.^5^ While the introduction of CBD is intended to improve overall surgical training, it is unclear what impact this approach will have on trauma training particularly given the evolution towards non-operative management.^2^

Unlike the ACGME Case Log System, the RCPSC does not require case log submission by trainees and does not have a national database to monitor such training parameters at individual resident or institutional levels. While most will agree that large case-volumes do not necessarily equate with competency,^6^ it is unreasonable to accept that surgical competency can be achieved without some basic minimum exposure.

We hypothesized that resident experience in operative trauma for general surgery residents in Canada is limited. The primary objective of our study was to determine the resident experience in operative trauma by the general surgery residents who completed their full residency training within the last decade at a single Level 1 trauma and academic health science centre. Secondary objectives were to determine the distribution of trauma surgery cases in terms of case mix, timing of operation, and surgical procedures performed.

## Methods

The study was a retrospective analysis of general surgery residents of McMaster University and their residency training at the Hamilton General Hospital (HGH) which is the sole source of mandatory trauma exposure during training. The HGH Trauma Registry was used to identify all trauma operations performed during the study period of July 1^st^, 2008 to June 30^th^, 2018. The trauma operations were then screened for inclusion in the study cohort by examining for the presence of an operative component identified within the RCPSC Surgical Procedure A or B Lists.^4^ Operations that met this inclusion criterion had their charts abstracted for types of operations and surgical procedures performed, the surgical service(s) present and the list of residents and fellows present. The names of the residents and fellows were cross-referenced with a complete list of all residents in each Post-Graduate Year (PGY) for the study period obtained from the General Surgery Residency Program office. Chart abstraction included the review of both the Operative Note(s) dictated by the surgeon(s) and the official Nursing Records. Demographic information was obtained from the Trauma Registry.

### Analysis

The resident role in the trauma operations was not directly documented in the hospital chart. An algorithm was developed by the study team to infer role (see Supplemental Digital Content). Start time of the operation was categorized as weekday (Monday to Friday, 8am to 5pm), weeknight (Monday to Thursday, 5pm to 8am) or weekend (Friday at 5pm to Monday at 8am). Data were analyzed using descriptive statistics. Categorical variables are expressed as frequencies and percentages, and continuous variables as means and medians. Differences between groups with regard to patient demographics, injury, and treatment measures were assessed using chi-square and Fisher's exact tests for categorical variables, and Student's t-test for continuous variables. All statistical analyses were carried out using SPSS version 25.0 (IBM Corp., Armonk, NY, USA). The study received ethics approval from the Hamilton Integrated Research Ethics Board and permission from the Residency Program.

## Results

The distribution of the start time and day of the week of any trauma operation revealed that over 75% of all trauma operations started after 5pm on weekdays or on weekends. The surgical specialty of attending surgeons present at trauma operations varied according to operation-type, with general surgeons present in 98.6% (416/422) of any-type abdominal operations, 74.8% (77/103) of any-type thoracic operations, and 46.7% (21/45) of any-type neck operation. General surgery was the sole surgical service involved in 81.3% (343/422) of any-type abdominal operations, 40.8% (42/103) of any-type thoracic operations, and 13.3% (6/45) of any-type neck operations.

The Hamilton General Hospital had a mean of 584 trauma activations per year during the study period, with blunt mechanism in 90.7% and penetrating in 7.5%. Average annual trauma operative volumes were 42 abdominal, 10 thoracic, 4.5 neck. General surgery residents were *not* present at 8% of abdominal operations, 35% of thoracic operations (which includes emergency department thoracotomies), and 62% of neck operations.

When analyzed for the best case and worst case resident operative role according to the *a priori* developed algorithm (see SDC 1), “primary operative” role could have occurred in abdominal operations at best up of 70% and at worst 0%, and was even less likely to occur for thoracic or neck operations.

**Figure 1:**
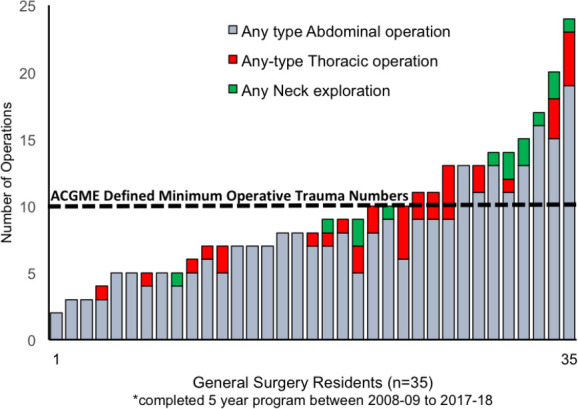
Distribution of operative exposure for graduating residents according to ACGME defined minimum operative trauma cases

A total of 35 residents successfully completed their entire general surgery residency training during the study period and their procedure-specific operative exposure is displayed in Table 1. A frequency-distribution histogram of all trauma operative exposures is presented in Figure 1 with the ACGME defined minimum operative trauma case number overlaid for comparison purposes.^7^ Only 14 of 35 (40%) of the residents met or exceeded the ACGME ten operative trauma case minimum requirement. Furthermore, only 10 (29%) were exposed to a neck exploration for trauma and only 18 (51%) were exposed to a thoracic operation for trauma.

**Table 1:** Trauma operative exposure among 35 general surgery residents who completed a full 5-year residency

	Total	Mean	Median
Index Laparotomy*	**213**	**6.09**	**5.0**
**Abdominal Intra-operative Procedure**			
Splenectomy	49	1.4	1.0
Liver Repair/Resection	18	0.5	0.0
Bowel Repair/Resection	58	1.7	1.0
Diaphragm Repair	37	1.1	1.0
Retroperitoneal Exploration	11	0.3	0.0
Pancreas Resection	5	0.1	0.0
Duodenal Repair/Resection	8	0.2	0.0
Renal Repair/Resection	1	0.0	0.0
Bladder Repair/Resection	6	0.2	0.0
Major Vascular Repair	9	0.3	0.0
**Thoracic Intra-operative Procedure**			
Cardiac Repair	2	0.1	0.0
Lung Repair/Resection	6	0.2	0.0
Major Vascular Repair	9	0.3	0.0
**Any-type Thoracic Operation**	**35**	**1.0**	**0.0**
ED Thoracotomy	4	0.1	0.0
OR Thoracotomy/Sternotomy	28	0.8	0.0
OR VATS	**3**	**0.1**	**0.0**
**Any-type Neck Exploration**	**15**	**0.4**	**0.0**

ED, Emergency Department

OR, Operating Room

* , includes non-therapeutic laparotomies

VATS, Video-Assisted Thoracoscopic Surgery

## Discussion

The RCPSC Objectives of Training document for the Specialty of General Surgery does not stipulate any quantitative volume of operative cases required to complete the residency requirements.^4^ However, it does stipulate a list of procedures that a graduate must be competent to independently perform which includes surgical exploration of penetrating neck injuries, resuscitative thoracotomy, and trauma laparotomy. The ACGME, which is the accrediting body in the USA equivalent to the RCPSC, describes specific operative case minimums (minimum of 10 trauma operations).^7^

The typical graduating McMaster resident was present for five index trauma laparotomies, zero thoracic trauma operations, and zero neck trauma operations. The vast majority of residents graduated the program seeing less than ten trauma operations, and indeed one resident graduated seeing only two trauma operations of any type. Given that all trauma operations do not provide equivalent procedural learning opportunities, our data shows the typical graduating resident will have been present for a total of one splenectomy, one traumatic bowel surgery, and one diaphragm repair, and this does not account for their operative role, which under optimal circumstances was likely “primary operator” in only 70% of such exposures.

There does not exist any other published Canadian data for comparison. However, these results raise questions about the resident competence to independently perform the required operations outlined by the RCPSC. Compared to US general surgery residency programs,^1^^,^^3^ McMaster University residents performed even fewer operative trauma cases and 60% of graduating residents would not satisfy ACMGE accreditation requirements.

Research into Canadian trauma training curriculum design demonstrates the major themes of institutional context and transferability of curricular components. Trauma volume, including direct operative experience, is highlighted as a critical aspect. Nevertheless, the identification of the value of transferable experiences and skills from non-trauma encounters is a promising avenue to improve overall training in trauma care.^8^ Further research in this area is sorely needed.

The limitations of our study include possible errors in documentation of resident presence and difficulty ascertaining resident role in the operation that we attempted to address with our interpretative algorithm. Strengths of our study include a full decade of study, use of two separate data sources to identify resident participation, and combined trauma registry and chart-level data abstraction methodology.

Some of the opportunities and challenges that exist to increase resident exposure to operative trauma are: 1) ensuring residents are present at all opportunities^9^ (e.g. use of in-house call versus home-call, augmenting after-hours exposures, ensuring general surgery participation even when sub-specialty surgical services become involved); 2) increase use of non-clinical simulation-based surgical technical skills courses^10^ and, 3) consider mandatory national or international rotations at high-volume trauma centres.^11^ Given the changing paradigm of general surgery training and practice,^12^ and in the footsteps of many other now-separate surgical specialties that originated within general surgery (e.g. thoracic surgery, vascular surgery, pediatric surgery, surgical oncology, colorectal surgery), the possibility of removing trauma competencies from the *mandatory* objectives of training of General Surgery and migrating such competencies to separate specialization (i.e. AFC Trauma General Surgery)^13^ should at least be contemplated.

## Conclusion

Our study represents the first ever publication of operative trauma case log volumes of a RCPSC General Surgery Residency Program. The results from our study provide a valuable description of the operative trauma exposure at a single academic healthcare network in Canada and importantly raise questions about the adequacy of the operative exposure to satisfy the competency requirements of training in trauma care. Based on these results, the Canadian Collaborative on Urgent Care Surgery (CANUCS) of the Canadian Association of General Surgeons is currently undertaking a nation-wide multi-centre study to further examine the actual operative, non-operative, and structured educational exposures occurring at general surgery training programs in Canada -- namely the Trauma RECON study (Trauma Resident Exposure in Canada and Operative Numbers).
